# Supplementary material for: Identification of male COPD patients with exertional hypoxemia who may benefit from long-term oxygen therapy
Source: PLoS One. 2023 Apr 6;18(4):e0283949. doi: 10.1371/journal.pone.0283949 (PMC10079074; doi:10.1371/journal.pone.0283949)
Supplement: Attachment — Submitted filename: Response to Reviewers PONE-D-22-26533.doc [file pone.0283949.s001.doc]

December 4, 2022

Emily Chenette, PhD

Editor-in-Chief,

*PLOS ONE*

Dear Dr Chenette,

Thank you for the opportunity to respond to these thorough reviews of our manuscript “**Identification of COPD patients with exertional hypoxemia who may benefit from ambulatory oxygen”** (Manuscript ID PONE-D-22-26533). We hope that our responses and edits to the manuscript will lead to the publication of this important manuscript.

Sincerely,

Michael Campos, MD, FACP

Corresponding author

RESPONSE TO REVIEWERS

We truly appreciate your critical review of our manuscript. Based on your comments, we have added new figures (showing how the study population was derived and a Kaplan Meier analysis). Table 1 was split in two tables to present our results more clearly.

**REVIEWER 1**
1. It’s important to be clear about the different types of supplemental oxygen therapy. The LOTT trial evaluated long-term oxygen therapy of 24-hour duration. This should not be considered equivalent to ambulatory oxygen therapy, which is used during exertion or activities only. The authors appear to have considered both as a similar therapy. This should be clearly differentiated throughout the paper with appropriate terminologies used. Please refer to the 2020 ATS guideline as guidance.

**Answer: We agree with the reviewer that there are important differences between our prescription of oxygen, and that of the LOTT trial. In the LOTT trial publication in the 2016 NEJM article they describe their prescription: Patients in the supplemental-oxygen group were prescribed 24-hour oxygen if their resting Spo2 was 89 to 93% and oxygen only during sleep and exercise if they had desaturation only during exercise. Our study population focused on subjects with exertional hypoxemia . We prescribed oxygen only during ambulation or exertion for all of these patients and did not encourage nocturnal use of oxygen. We added these important differences to the Discussion.**

2. The main reason for different recommendations on the use of ambulatory oxygen therapy is the low level of evidence in this area. This should be emphasized.

**Answer: We agree with this statement and have emphasized it in the Discussion as the first line in the last paragraph.**

3. Methods, study population: The definition of exertional desaturation should be moved to this section, instead of being part of the 6MWT protocol. There is a need to clarify the definition of exertional desaturation – was an arterial line inserted as part of the assessment? Otherwise, how did the authors evaluate the presence of a drop of PaO2 to < 55mmHg for at least 15s?

**Answer: We have moved the definition of exertional desaturation as recommended. Exertional hypoxemia (drop for at least 15 seconds) was defined with continuous SpO2 monitoring in all subjects. The majority had an ABG immediately done after documentation of hypoxemia at the end of minute 6, but not all. We see that this may cause confusion to the reader and since this final ABG did not affect the selection of the population or definition of the group, we have omitted it from the manuscript and Table.**

4. One of the major limitations of this study is the lack of measurements of oxygen usage. With the provision of a stationary concentrator for home use, patients could have been using them at rest as well.

**Answer: That is true and as noted in the Discussion and is difficult to evaluate in a real-life study. This non-adherence with prescribed oxygen therapy likely occurred in subjects from both groups, and we are not able to assess differences in hours of oxygen use between groups in our study.**

5. For consideration of immediate responses in 6MWD for supplemental oxygen therapy, how were those who had exactly improved 6MWD by 54m considered?

**Answer: No subjects improved exactly 54m.**

6. Statistical analyses: With regards to the Cox regression, how were patients who received lung transplantation, had lost to follow-up, or progressed to resting hypoxaemia being considered? Also which FEV1 was used for the adjusted analyses, which may affect the findings? Were other prognostic risk factors being considered in the adjusted analyses?

**Answer: We repeated the survival analysis censoring on the date patients either progressed to severe resting hypoxemia on annual re-evaluation (N=34) or were referred for lung transplantation (N=1) and added comorbidities (Charlson Index) as a covariate. The mortality differences remained significant after these changes were made to the survival analysis. We used pre-bronchodilator FEV1 as most patients with long standing COPD only had pre-bronchodilator testing during follow-up. All this has been detailed in the new manuscript version.**

7. Results: It could be helpful to include a study flow diagram to provide details of patients considered and included in this study.

**Answer: Added as Figure 1 now.**

8. Results: There were no data on COPD medications and history of acute exacerbations, which are important as both can affect health outcomes of patients with COPD. Did any patients progress to resting hypoxaemia needing long-term oxygen therapy of ≥15 hours?

**Answer: We added medication use and progression to severe resting hypoxemia in the updated Table 1. There were no differences between the groups in these parameters.**

9. Results, Figure 1: It is more appropriate to present the Kaplan-Meier curve of responders and non-responders, which demonstrates the true / crude survival, rather than the adjusted Cox regression.

**Answer: This has been added and shown as Figure 2A.**

10. Discussion: As mentioned, causal relationships and reasons for improved survival noted in the responders could not be evaluated in this study. The sentence “improved survival in this group results from a gradual improvement in overall functional status” should be toned down and re-worded.

**Answer: It has been reworded and now reads: “We can only speculate that the improved survival in this group may have resulted from a gradual improvement in overall functional status, which needs further exploration in a prospective trial”.**

Minor comments
1. Abstract, sentence #1: This sentence appears to be missing the treatment, i.e. oxygen therapy.

**Answer: Thank you for noticing this omission.**

2. Abstract, Results: it is unnecessary to report up to 3 decimal places for the results of Cox regression

**Answer: Updated.**

REVIEWER 2

1. First, the authors found that responders, defined as subjects who improved their 6MWD more than 54 m under oxygen supplementation, had better survival compared to non-responders. Since responders had a lower 6mwt distance at baseline, these results might also suggest that a low distance 6MWT is predictive of longer survival. I believe that this needs better explanation.

**Answer: We are not stating that a low 6MWT predicts longer survival. We are suggesting that low functional capacity is a characteristic of subjects that may exhibit an immediate response to supplemental oxygen therapy by increasing walk distance, and if so, associated with improved survival. The association of low 6MWT with immediate response to O2 was observed by Jarosch et al (Chest. 2017;151(4):795-803) but the association with mortality in a real life group of subjects is novel.**

2. A selection bias might be possible. Within the seven-year period they recruited about 160 patients. This number seems rather low. How the authors selected their data? Was there a database/ Did they use certain criteria applied? Who has extracted data? Which rule applied?

**Answer: As can be noted in the new Figure 1 (flow chart), we selected patients from the entire population who underwent home oxygen testing. This is an electronic list kept in the PFT lab records. We then performed chart reviews to narrow the population using strict criteria for COPD diagnosis. Other reasons to exclude patients are noted in the flow chart. We were surprised that the number in the two groups were similar.**

4. Echocardiography performed closest to the evaluation – was the time delay between assessment and 6mwt similar in both groups?

**Answer: Yes, as with other parameters we analyzed, echocardiography was performed within one year of the date of the 6MWT.**

5. The relationship between the type of response and mortality may have not accounted factors that may have also contributed to survival. i.e. treatment? Participation in rehabilitation programs? Other diseases? Years of disease? In this respect an additional survival analysis might be helpful.

**Answer: As noted in the updated Table 1, we have added medication use, progression to severe resting hypoxemia, referral to pulmonary rehabilitation and did not find differences between the groups. Comorbidities were added in the Cox analysis without change in the mortality result**

Minor comments
Abbreviated Lung function tests in Table 1 may need explanation

**Answer: Done. Thank you.**
