# Supplementary material for: Identification of male COPD patients with exertional hypoxemia who may benefit from long-term oxygen therapy
Source: PLoS One. 2023 Apr 6;18(4):e0283949. doi: 10.1371/journal.pone.0283949 (PMC10079074; doi:10.1371/journal.pone.0283949)
Supplement: Attachment — Submitted filename: Response to Reviewers PONE-D-22-26533R1.doc [file pone.0283949.s002.doc]

January 22, 2023

Emily Chenette, PhD

Editor-in-Chief,

*PLOS ONE*

Dear Dr Chenette,

Thank you for the opportunity to respond to these thorough reviews of our manuscript “**Identification of COPD patients with exertional hypoxemia who may benefit from ambulatory oxygen”** (Manuscript ID PONE-D-22-26533R1). We hope that our responses and edits to the manuscript will lead to the publication of this important manuscript.

Sincerely,

Michael Campos, MD, FACP

Corresponding author

RESPONSE TO REVIEWERS

We again truly appreciate your critical review of our manuscript. Hopefully this time we have addressed your concerns to your satisfaction.

**REVIEWER 1**
Thanks for the revised manuscript. Most comments have been addressed adequately, with only a couple minor ones remaining.
1. Need to add the number at risk to the bottom of the survival curves (Figure 2)

ANSWER: The Figure has been updated.

2. Table 1: Need to add how were the annual severe exacerbation rates evaluated (e.g. during the previous 12 months prior to assessment? from diagnosis? or others)

ANSWER: The annual exacerbation rates were evaluated prospectively since the day of the 6MWT (labelled as ‘during follow up’ in Table 1). We had added this explanation in the Methods section.

**REVIEWER 2**
The authors have responded to my comments, but I have still concerned regarding their response.

1. It was asked to the authors to explain if their results might also suggest that a low distance 6MWT at baseline is also predictive of longer survival (and how?). The same question arises if someone asks how a group of COPD patients with increased BODE score, presents better survival compared to a group with lower BODE index. I think this is the case in this article. To my view this question has not been adequately explained.
ANSWER: We apologize for the insufficient explanation. We would like the reviewer to know that this surprising observation prompted us to write this manuscript. We had observed that some patients with exertional hypoxemia walked more than others when placed on oxygen therapy, but we did not expect to see significant differences in survival. The mechanism by which this occurs can only be hypothesized at this point. As the reviewer points out, possibilities might be explained by differences in oxygen uptake by lungs (V/Q mismatch) or even muscle, or differences in oxygen delivery. Our observational cohort was not designed to assess this and that is why not all patients had an echocardiogram at the time of the walk test or more complete cardiopulmonary exercise testing. Echocardiograms were reviewed to assess if patients had pulmonary hypertension that prompt us to categorize them as having severe resting hypoxemia at a higher oxygen saturation. We believe that at this time the most important reason to publish our observations is to bring awareness to other COPD researchers that there may be an important subgroup of patients with exertional hypoxemia in whom oxygen therapy may have benefits that are previously unreported including prolonged survival. We also want to notify the reviewer that we are using our data as the basis for a new prospective study to answer exactly the questions he is asking us. This will include complete metabolic stress testing, V/Q scan, echocardiography including non-invasive assessment of pulmonary vascular resistance, and evaluation of sarcopenia for responders and non-responders. Responders will then be randomized to receive supplemental oxygen or sham oxygen prospectively with a primary goal of monitor changes in daily activity using actimetry, but also performing cardiopulmonary exercise testing and echocardiography with and without oxygen to better understand potential mechanisms of improved exercise capacity. It is possible that BODE scores improve in responders on oxygen due to increases in their 6MWD. Within the limits of our current understanding of this phenomenon, we have expanded our explanations in the Discussion, hopefully to the reviewer’s satisfaction.

Comment 2. Echocardiography was performed within one year of the date of the 6MWT. The question was if the delay between Echo- and 6MWT was similar in the two groups. The authors have not added anything in the text (either in the results or in discussion as a potential limitation).

ANSWER: As mentioned above, echocardiograms were ordered clinically not in relation to the study or decision to prescribe oxygen. We decided to review them to ensure correct oxygen prescription with a higher SpO2 threshold if the subject had pulmonary hypertension and at the same time assess if that factor influenced the walking response. Since not all subjects had an echo and timing was not uniform in the group, we agree is a limitation that we have now added to the Discussion.

Comment 3. It was asked to the authors to provide some details about the method of data acquisition. They refer to Figure 1 (Flow chart). However, I do not see any description about the database in the text.

ANSWER: We had added a brief description of the flow chart in the Methods Section, as most of the information regarding subject exclusion is detailed in the Figure.

Comment 4. 2. In survival analysis, variables (Charlson index, FEV1 and age) which were not different between groups in univariate analysis have been accounted in. A rational for the variables entered in COX analysis might be helpful for the reader. I asked to the authors about factors that may have also contributed to survival. namely: treatment, participation in rehabilitation programs, other diseases, Years of disease. There is no data on the duration of the COPD and the co-existence of coronary/ischemic disease that could explain differences in functional tests.

ANSWER: Our choice of covariates to adjust our survival analysis was based on the Guidance for Authors from Editors of Respiratory, Sleep, and Critical Care Journals published in 2019 by Lederer et al. (1). In this publication it is highly recommended that covariates need to be chosen by clinical grounds (“historic confounders”) and not by extrapolation of statistically significant factors found randomly after performing large comparison of factors between groups (p-value based methods). For that reason, we chose age and FEV1 (a surrogate of severity, duration of COPD and known factor that affects survival). The reviewer asked about adjusting for comorbidities and we sought this was not unreasonable as mortality is affected by increased number of comorbidities in COPD (2, 3). We therefore used the Charlson’s comorbidity score in the model even if it was not different between groups. The observed mortality difference between responders and non-responders persisted. Following the Guidance recommendation, we do not think that it is appropriate to further divide individual comorbidities for the model at this point. As the reviewer is concerned with heart disease and the effect of cardiac output, we looked at the proportion of individuals diagnosed with heart failure (already included in the Charlson’s Index) and noted that it was present in 22% in responders and 25% in non-responders (NS). Similarly, when reviewing the available echocardiograms, we noted that heart failure could be ascribed to 16% of responders and 18% of non-responders (NS). We have added these comments in the manuscript, hopefully now to the reviewer satisfaction.

Regarding referral to pulmonary rehabilitation, it was added to the bottom of Table 1 as per the reviewer’s initial request. We do not have access to document rehab compliance in this retrospective analysis. The duration of COPD is difficult to assess as COPD is a condition of gradual onset. We rely on FEV1 as a marker of severity. COPD therapies were applied equally in both groups.

**REFERENCES**

1.	Lederer DJ, Bell SC, Branson RD, Chalmers JD, Marshall R, Maslove DM, et al. Control of Confounding and Reporting of Results in Causal Inference Studies. Guidance for Authors from Editors of Respiratory, Sleep, and Critical Care Journals. Annals of the American Thoracic Society. 2019;16(1):22-8.

2.	Sin DD, Anthonisen NR, Soriano JB, Agusti AG. Mortality in COPD: Role of comorbidities. The European respiratory journal. 2006;28(6):1245-57.

3.	Mannino DM, Thorn D, Swensen A, Holguin F. Prevalence and outcomes of diabetes, hypertension and cardiovascular disease in COPD. The European respiratory journal. 2008;32(4):962-9.
