# Supplementary material for: Identification of male COPD patients with exertional hypoxemia who may benefit from long-term oxygen therapy
Source: PLoS One. 2023 Apr 6;18(4):e0283949. doi: 10.1371/journal.pone.0283949 (PMC10079074; doi:10.1371/journal.pone.0283949)
Supplement: Attachment — Submitted filename: Response to Reviewers PONE-D-22-26533R3.doc [file pone.0283949.s003.doc]

March 18, 2023

Emily Chenette, PhD

Editor-in-Chief,

*PLOS ONE*

Dear Dr Chenette,

Thank you for the opportunity to respond again to the minor edits required for the acceptance our manuscript “**Identification of COPD patients with exertional hypoxemia who may benefit from ambulatory oxygen”** (Manuscript ID PONE-D-22-26533). We hope that this last set of responses and edits to the manuscript will lead to its publication.

Sincerely,

Michael Campos, MD, FACP

Corresponding author

RESPONSE TO REVIEWERS

We again truly appreciate again your critical review of our manuscript. Hopefully this time we have addressed your concerns to your satisfaction.

Reviewer 1 agreed to accept the second revised version of the manuscript.

1) I think the mechanism by which COPD patients with exertional hypoxia and poor 6MWD were more benefited by long-term oxygen treatment seems plausible after carefully reading the authors' comments to Reviewer 2. The responses to comments 2 and 3 also seem relevant. Regarding comment 4, the CCI adjustment in the multi-variable analysis appears appropriate.

**Response:** We are glad our explanations are felt as sound as the findings are of clinical relevance and hypothesis generating to be explored in a prospective study.

2) I like to propose an additional sensitivity analysis in patients who are men. I noticed that most of the patients who have been enrolled are men. Perhaps the research title might be changed to "Identification of male COPD patients" if the authors conducted a re-analysis on male patients. This would be more informative for the readers.

**Response**: We had followed this suggestion and excluded 22 women from the initial COPD dataset, including 4 from the population analyzed. It did not affect the overall results. We had edited all the tables, statistics and Figures.

3) In addition, as Reviewer 2 first suggested, I recommend describing the dataset and patient selection process in the main text in more detail.

**Response**: This has been done.
